# Expression Level of IL-17 in Peripheral Blood of Patients with Late Pregnancy and Diagnosis of Maternal-Fetal Tolerance Based on Brain MRI Image Segmentation Algorithm

**DOI:** 10.12669/pjms.37.6-WIT.4828

**Published:** 2021

**Authors:** Zenying Yu, Shengyan Zhou, Zhen Tan, Guangmin Lu

**Affiliations:** 1 Zenying Yu, Bachelor’s Degrees. Department of Gynaecology and Obstetrics, The Third People’s Hospital of Linyi, Linyi 276023, China; 2 Shengyan Zhou, Bachelor’s Degrees. Department of Gynaecology and Obstetrics, Lanling County People’s Hospital of Linyi, Linyi 277000, China; 3 Zhen Tan, Master of Medicine. Department of Pathology, The Third People’s Hospital of Linyi, Linyi 276023, China; 4 Guangmin Lu, Bachelor’s Degrees. Department of Endocrinology and Metablism, The Third People’s Hospital of Linyi, Linyi 276023, China

**Keywords:** Peripheral blood, IL-17, Treg cells, Image Segmentation Algorithm, MRI

## Abstract

**Objectives:**

To study the expression of IL-17 in peripheral blood and its effect on maternal-fetal tolerance in patients with eclampsia in late pregnancy using MRI image segmentation algorithm.

**Methods:**

Thirty-nine patients with severe preeclampsia and eclampsia with brain symptoms were examined by cranial MRI. Pregnant women with 32 weeks of pregnancy were selected to detect the percentage of Th17 and Treg cells in CD4 + T lymphocytes and the expression of cytokines IL-17 and IL-10 in peripheral blood.

**Results:**

MRI examination was normal in 26 cases, 9 cases showed reversible posterior encephalopathy syndrome, three cases were cerebral hemorrhage, and one case was intracranial cavernous sinus thrombosis. two. Compared with the mild preeclampsia group, the relative number of Thl7 cells increased and that of Treg cells decreased in the severe preeclampsia group (P>0.05).

**Conclusion:**

The major types of cerebrovascular diseases (CVD) in severe preeclampsia and eclampsia were reversible posterior encephalopathy syndrome and cerebral hemorrhage. It was speculated that the damage to the blood-brain barrier may play an important role in the pathogenesis. The balance of the number of Th17 cells/the number of Treg cells was more inclined to the Th17 cell-mediated pro-inflammatory state, Treg cell-mediated immune tolerance decreases, and it becomes more obvious with the worsening of the disease.

## INTRODUCTION

Preeclampsia (PE) is a common pregnancy complication, usually involving multiple organ functions and a series of maternal and infant complications. It is found that immune factors are closely related to the occurrence and development of PE.^1^ Treg cells can induce the formation of immune tolerance, make pregnancy progress smoothly, and have unique immunoregulatory functions. Thl7 can mediate inflammatory responses and secrete interleukin 17 (IL-17), interleukin 22 (IL-22) and other cytokines. Therefore, Thl7 and Treg cells may be involved in embryo immune rejection and inflammation of pathological pregnancy such as PE.^2^

Reversible posterior leukoencephalopathy syndrome (PRLS) is the neuroradiological syndrome, with headache, blurred vision, and confusion as the main clinical manifestations. Most patients can be cured, but untimely or ineffective treatment can aggravate the condition, endangering the lives of patients. Therefore, early diagnosis of patients is necessary.

Above, Th17 may be involved in the embryonic immune rejection and inflammatory response of PE. It can mediate the inflammatory response and secrete interleukin 17 (IL-17) and other cytokines, but the correlation between the expression of IL-17 in peripheral blood and maternal-fetal tolerance of PE patients remains unclear. In this study, 39 pregnant women with severe PE who underwent cranial MRI examinations were taken as the research subjects, to explore the relationship between the expression of IL-17 in the peripheral blood and the mother-fetal tolerance of PE patients, expected to provide a reference basis for the diagnosis and treatment of patients with eclampsia in late pregnancy.

## METHODS

There are many strategies for color and texture feature fusion. The brain MRI image segmentation algorithm uses the implicit fusion of color texture. Gabor filter is to extract feature vectors on each color channel, and then the Gabor feature vectors on the three channels are combined into the final feature vector. A color channel is taken as an example to briefly explain the method of extracting pixel texture feature values using a Gabor filter.^3^,^4^

Let *I*(*x,y*) and (*x,y*) ∈ Ω represent the set of all pixels of the texture feature image to be extracted. It is a Gabor feature image obtained by performing a convolution operation with a 2D Gabor filter *g*(*x,y*):


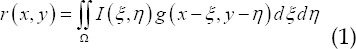


Among them, *g*(*x,y*) is a linear filter, and its impulse response is realized by multiplying the Harmonic function by the Gaussian function in the space range. According to Dagupan’s definition in the literature, this Gaussian function is given:


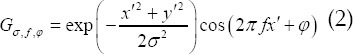



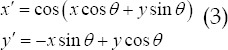


*θ* is the angle parameter, *f* is the frequency parameter, and *ϕ* is the phase offset? *ϕ* is often set to 0 to implement even symmetrical filters. The parameter *σ* determines the support area of the Gabor function, and the parameters ^*f*^ and *σ* determine the bandwidth b of the spatial frequency.


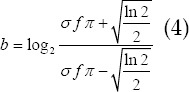


The Gabor filter is a band-pass filter. The parameters *f, ϕ* and *σ* determine the sub-bandwidth. In the whole space-frequency domain, the Gabor filter can extract different features that meet specified conditions in the image by setting different parameter combinations.^5^

Twenty-three pregnant and maternal patients with severe preeclampsia and eclampsia admitted to the obstetrics department of our hospital were selected. The diagnosis of hypertension during pregnancy was based on the 7th edition of Obstetrics and Gynecology. The diagnosis of CVD was examined by MRI, and the diagnosis was based on the revised standards of the 4th National Cerebrovascular Disease Conference of the Chinese Medical Association. The general information, clinical symptoms, blood pressure, and laboratory results of the two groups of patients were compared. The highest blood pressure value measured within 6 h before the onset of seizures is selected. The blood pressure change value is the difference between the highest blood pressure and the basal blood pressure measured within six hours before the onset of disease. Laboratory tests include hemoglobin (Hb), hematocrit (HCT), platelet count (PLT), aspartate aminotransferase (AST), alanine aminotransferase (ALT), alkaline phosphatase (ALP), lactic acid Dehydrogenase (LDH), blood creatinine (Cr), blood uric acid (UA), blood urea nitrogen (BUN), plasma albumin (Alb), 24h urine protein monitoring after admission were the highest quantitative values.^6^

Blood samples were reserved at 32 weeks of pregnancy. All patients signed informed consent.

### Inclusion criteria:

- Han nationality;
- Aged 24 to 34 years;
- Single pregnancy;
- No threatened labor symptoms.

###  Exclusion criteria

complications during pregnancy (such as thrombocytopenia during pregnancy, gestational diabetes, pregnancy anemia, intrahepatic cholestasis of pregnancy); previous hypertension, heart disease, diabetes, glaucoma, epilepsy, autoimmune disease; fetal growth restriction; severe bacterial or viral infectious diseases (upper respiratory infections, infectious diarrhea) in the last one month; taking immunosuppressants in the last six months.

Pipette tips, centrifuge tubes, coverslips, plastic pipettes, flow loading tubes, sodium heparin anticoagulation tubes, Eppendorf tubes of various specifications (EP tubes), micropipettes (Thermo Fish); 5% CO_2_ incubator (NV2500E, American Heraeell Company); Ultra-low temperature refrigerator (Japan Mitsubishi Corporation); 4 ° C, -20 ° C refrigerator (Qingdao Haier Group); Super workbench (Suzhou Purification Equipment Factory); desktop high-speed centrifuge (Shanghai Medical Instrument Factory); BP-211D electronic analytical balance (Sartorius, Germany); inverted phase contrast microscope: OlympusIMF-2 (Olympus, Japan); FACS Calibar flow cytometer (BD Bioscience, Canada) Pharmingen).

All selected patients were examined by MRI of the brain. First, the axial and sagittal examinations were performed with T1WI and T2WI synchronously. Patients were subjected to diffusion-weighted imaging examinations. Neuroradiologists confirmed the diagnosis of intracranial venous sinus thrombosis, cerebral infarction, and reversible PRLS. Relevant diagnostic criteria revised by the Cerebrovascular Disease Academic Conference are in line.

Statistical software (SPSS17.0) was used for statistical analysis. Measurement data was expressed by (*x̅*±*s*), tested by t value, counted data was expressed by (%), and χ^2^ test was used. Statistical significance.

## RESULTS

There were 14 patients with eclampsia, 12 patients had negative MRI results, and 28 patients had positive MRI results, including one case of intracranial venous sinus thrombosis, one case of cerebral infarction, and 26 cases of refolding PRLS. MRI findings of patients with reversible PRLS showed bilaterally symmetrical lesions as the main feature, and cortical and subcortical white matter edema as the main features. The lesion T1WI showed slightly lower and lower signals, T2WI showed slightly higher signals, and diffusion-weighted imaging showed slightly higher signals.

There were no statistical differences in the general conditions of age, onset of gestational age, parity between the two groups.

There was no statistically significant difference in diastolic and systolic blood pressure at the onset of disease between the two groups, P>0.05; the systolic pressure change and mean arterial pressure in the research group were higher than those in the control group, *P<*0.05.

The serum uric acid level in the control group was lower versus the research group, showing notable differences (*P<*0.05). There was no statistical comparison of red blood cell volume, platelet count, blood calcium, blood urea nitrogen, alkaline phosphatase, hemoglobin, plasma albumin, blood creatinine, aspartate aminotransferase, and alanine aminotransferase in the two groups, P>0.05. Table-I.

**Table-I:** Comparison of laboratory indicators between the two groups (*x̅*±*s*).

*Group*	*Serum uric acid (mmol/L)*	*Hematocrit (%)*	*Platelet count (× 109/L)*	*Blood calcium (mmol/L)*	*Blood urea nitrogen (mmol/L)*
Control group	242.6±57.4	32.6±3.1	195.8±57.2	2.1 ±0.2	4.9±1.6
research group	335.8±72.6	34.4±5.9	166.6±73.5	2.1±0.6	4.2±2.8
P	<0.05	>0.05	>0.05	>0.05	>0.05

*Group*	*Alkaline phosphatase (U/L)*	*Hemoglobin (g/L)*	*Plasma albumin (g/L)*	*Serum creatinine (mmol/L)*	*Aspartate aminotransferase (U/L)*

Control group	155.5±44.4	107.8±13.8	29.6±4.5	59.6±14.1	20.9±12.7
research group	133.9±52.8	115.8±20.5	26.8±6.4	78.8±43.9	89.8±76.4
P	>0.05	>0.05	>0.05	>0.05	>0.05

## DISCUSSION

The risk factors of PE and eclampsia are to some extent the risk factors of PRLS, arising from physiological changes during pregnancy.^7^ The results of this study found that, in the control group, the changes in systolic blood pressure and mean arterial pressure were more obvious (*P<*0.05). It suggested that, elevated blood pressure was an important factor in the formation of PRLS. Mueller-Mang et al. (2009) proposed that, the degree of blood pressure elevation is closely related to the PRLS patient’s basal blood pressure. If the basal blood pressure rises significantly, even if the blood pressure is normal, it will develop into PRLS.^8^ Additionally, the serum uric acid level in the control group was significantly lower than that in the research group (*P<*0.05). Studies have pointed out than 9 and the level of 24h serum uric acid in women with severe PE was significantly higher than the group without RPLS (*P=*0.012).^10^

Th17 cells increases significantly when the body undergoes an enlarged inflammatory response. The initial CD4 + T cells can produce specific IL-17 and secrete IL-6 and IL-21. Cytokines participate in inflammatory reactive diseases of the body by aggregating and activating neutrophils before releasing inflammatory factors.^11^ IL-17 can maintain CD34+ proliferation of hematopoietic progenitor cells and cause infiltration and destruction of tissue cells. Th17 cells and its cytokine IL-17 are important in the pathogenesis and progression of PE.^12^ Higher IL-17 can aggravate the inflammatory response on the inner wall of small blood vessels of the placenta, release a variety of active substances, damage the vascular endothelial cells, and cause patients with placenta and small bodies Vascular spasm, lead to the occurrence of PE.^13^ Darmochwal-Kolarz et al. (2012) pointed out that, the increased expression of IL-17 in peripheral blood and placenta tissue of PE patients may be associated with the increase of Th17 cells.^14^ There were investigators using flow cytometry to assess the proportion of Thl7 cells in the peripheral blood. It was found that, its level was significantly increased in PE pregnant women, which was associated with the increase of IL-17 mRNA level in the decidua tissue of PE women.^15^ The results of this study showed that, compared with the normal pregnancy group, the mild and severe PE groups showed significantly increasedTh17/CD4+, Treg/CD4+, and Log/Treg content (*P<*0.01); among them, the increase of *Log/Treg* relative cell number in the severe PE group was more obvious (*P<*0.05). These results indicated that, the increase in Th17 cells was associated with the occurrence and development of eclampsia, which further validated the hypothesis of Darmochwal-Kolarz et al^14^ Plus, overexpressed Th17 cells can’t embryo implantation, or superficial implantation occurs, leading to pregnancy complications.^16^ In PE patients, the decrease of Treg cells reduces the body’s immune tolerance, and the increase of Th17 cells enhances the body’s immune response. Together, the two have led to the development of PE and the severity of the disease.^17^

Pregnant women undergoing MRI examinations have not experienced short-term, mid-to-long term complications, or adverse reactions. The purpose of MRI is not only to show the condition of the lesion, but also to make a differential diagnosis. Patients without seizures may already have reversible PRLS. If the symptoms of encephalopathy are caused by infarction, bleeding, venous thrombosis the diagnosis is likely to cause treatment errors.^18^ According to the results of this study, compared with the control group, patients with reversible PRLS have diastolic blood pressure changes, mean arterial pressure changes, and increased blood uric acid. Based on this, we can recognize that reversible PRLS the clinical characteristics and perinatal outcomes of the patients were improved. The imaging changes of the restorable PRLS are mainly in the posterior occipital lobe of the cerebral hemisphere. Angioedema is the main pathological change.^19^ In clinical practice, reversible PRLS is prone to misdiagnosis, such as encephalitis. This is mainly due to the similarities between the two diseases and the clinical manifestations. Misdiagnosis is prone to occur, for which the differential diagnosis needs to be done.

### Limitations of the study

The etiology and pathogenesis of PE remained undetermined, which is characterized by varying clinical manifestations, complex pathological changes, and multi-system and multi-organ involvement. The results of this study indicated that, IL-17 plays a certain role in the pathological process of PE, and Th17 cells and Treg cells mediate the occurrence of PE. However, the related factors, signal pathways, and specific regulatory mechanisms involved in the occurrence of PE by IL-17 and Th17 cells need to be further studied.

## CONCLUSIONS

Th1/Th2, Th17, Treg cells and their cytokines form a complex immune network, and each factor regulates each other to keep the body in a sophisticated and complex immune balance state at any time. By studying the role of two subpopulations of Th17 and Treg cells and their related cytokines (IL-17, IL-10) in the pathogenesis of PE, it has made up for the lack of previous research. For patients with severe preeclampsia and eclampsia, reversible PRLS is the main characteristic damage after the occurrence of encephalopathy, which may be related to endothelial cell damage and damage to the blood-brain barrier. Through MRI examination, Analysis of the examination characteristics and clinical conditions can effectively diagnose severe preeclampsia and reversible PRLS, which has significant clinical value.

### Authors Contribution:

**SZ:** Conceived the study, literature review, participated in its design, coordination, analyzed the data and helped to draft the manuscript and also the responsible and accountable for the accuracy or integrity of the work

**ZT & GL:** Helped in design, data collection, article drafting & critical revision.

**ZY:** Takes the responsibility and is accountable for all aspects of the work in ensuring that questions related to the accuracy or integrity of any part of the work are appropriately investigated and resolved.
